# Preoperative anxiety and associated factors among women admitted for elective obstetric and gynecologic surgery in public hospitals, Southern Ethiopia: a cross-sectional study

**DOI:** 10.1186/s12888-023-05005-2

**Published:** 2023-10-09

**Authors:** Abera Mamo Dibabu, Teklemariam Gultie Ketema, Maechel Maile Beyene, Dereje Zeleke Belachew, Hailegiyorgis Geleta Abocherugn, Abdu Seid Mohammed

**Affiliations:** 1 Department of Midwifery, College of Medicine and Health Sciences, Mizan Tepi University, Mizan Teferi, Ethiopia; 2 Department of Midwifery, College of Medicine and Health Sciences, Arba Minch University, Arba Minch, Ethiopia

**Keywords:** Anxiety, Elective, Gynecologic, Obstetric, Preoperative, Women

## Abstract

**Background:**

Preoperative anxiety is a major mental health problem during the preoperative period. Admission of women to surgery is stressful, and a high level of anxiety was associated with increased perioperative morbidity and mortality, poor treatment satisfaction, and bad obstetric outcomes, including long-term cognitive impairment in children. Despite its negative consequences, little is known on this area, particularly in the study area.

**Objective:**

To assess preoperative anxiety and associated factors among women admitted for elective obstetrics and gynecologic surgeries in public hospitals in Southern Ethiopia, 2022.

**Methods and Materials:**

An institution-based cross-sectional study design was conducted among 389 women using structured interviewer-administered samples who were selected by systematic random sampling from May 20^th^ to June 20^th^, 2022. The Amsterdam preoperative anxiety and information scale (APAIS) was used to assess the level of anxiety. Data were collected electronically using the Open Data Kit version 2022.2.3 and analyzed with the Statistical Package for Social Sciences version 26.0. Bivariate and multivariable logistic regression analyses were done. The strength of the association was declared by using an adjusted odds ratio (AOR) with a 95% confidence interval, and a statistical significance of *P* < 0.05.

**Results:**

The magnitude of preoperative anxiety was 57.1% (95% CI = 51.4–61.5), and 48.1% of women required an average amount of information. Being a gynecologic patient (AOR = 2.0, 95% CI = 1.21, 3.32), having previous anesthesia and surgery (AOR = 2.09, 95% CI = 1.10, 3.96), having fear of postoperative pain (AOR = 1.96, 95% CI = 1.08, 3.53), having concern for family (AOR = 2.56, 95% CI = 1.49, 4.37), having poor social support (AOR = 3.75, 95% CI = 1.99, 7.09), and moderate social supports (AOR = 3.27, 95% CI = 1.74, 6.17), and having a high information requirement about anesthesia and surgery (AOR = 4.68, 95%CI = 2.16, 10.13) were statistically associated with preoperative anxiety.

**Conclusion:**

Preoperative anxiety was often high in the region. Associated factors were the type of surgery, previous anesthesia and surgery, fear of postoperative pain, fear for family, social support, and a high information need. So the national and regional health bureau should develop guidelines and implement strategies to reduce women preoperative anxiety as part of midwifery care. The women should be assessed regularly during the preoperative visits; and appropriate anxiety reduction and information regarding surgery, and anesthesia should be provided.

## Background

Anxiety is an unpleasant emotional state, including tension, apprehension, uneasiness, worry, and autonomic nervous system activation, which is a personal feeling resulting from a patient's reaction to the uncertainty of particular stimuli [1]. It is a rational and normal reaction to actual and potentially dangerous circumstances as a result of sympathetic, parasympathetic, and endocrine activation; however, excessive and prolonged worry leads to serious, life-threatening complications and increases post-operative morbidity and mortality [2]. Preoperative anxiety is a common reaction in patients admitted to surgery and is caused by "unknown, unsuccessful recovery and surgical failure, anesthesia-related fear, personal identity loss, fear of postoperative pain, a sense of loss of control, and a fear of death" [3, 4].

From conception of their fetus to completion of childbirth via cesarean section (CS) and various gynecologic (GYN) surgeries, which involve the organs and structures of the female pelvic region, including the reproductive organ, women face numerous challenges throughout their lives [5]. GYN surgery, more than other surgeries, evokes a variety of psychological and emotional issues due to loss of fertility, sexuality, and attractiveness, which are done for an indication of benign or malignant disease and are invasive procedures performed through an incision of the body that lead to bleeding, pain, and sometimes mortality [6].

The burden of mental illnesses such as anxiety and depression is greater than any type of chronic illness [7]. One in five women is affected by mental health problems such as anxiety, depression, and self-harm associated with life events and hormonal changes [8]. Hospitalizing a woman for surgery is a stressful event that leads to anxiety, which manifests itself in physiological, psychological, and cognitive responses [9].

Anxiety can be measured in many ways. It can be measured directly by measuring plasma cortisol and urinary catecholamine, or indirectly by measuring blood pressure and pulse [10]. Even if preoperative anxiety is a subjective phenomenon, the Amsterdam Preoperative Anxiety and Information Scale (APAIS) was specially developed for preoperative patients to measure the level of anxiety and the need for information on surgery and anesthesia [11].

The magnitude of preoperative anxiety among surgical patients differs in different countries' reports and operations types are as high as 92.6% [12] and also preoperative anxiety was highly observed in women in obstetrics and gynecologic procedures which accounts Thailand 23.2% [13], India 55% to 63.54% [14, 15], England 67% [16], Pakistan 72.7% [17], Nepal 51.81% [18] Sir Lanka 40.6% [19]. Whereas preoperative anxiety among women admitted for obstetrical surgeries in Ethiopia accounts 63% [20].

As compared to the general population, females and patients admitted for OB and GYN surgeries suffer more preoperative anxiety than any other types of surgeries like urologic and cardiac surgeries and male patients [21], which accounts 23.2%(13) to 63.45%(14). Even though the World Health Organization (WHO) recommended the optimal rate of cesarean section between 5 and 15% [22], the overall prevalence of CS in Ethiopia was 29.55 which poses permanent hazards to the mother, newborn, breastfeeding practice, and high prevalence of preoperative anxiety, which accounts for 55% to 72.7% [15, 17], and 63% in Ethiopia [20].

The impact of undiagnosed and untreated preoperative anxiety among women increases the odds of perioperative complications that lead to acute or long-term impact; some of them are difficult postoperative pain management, increased hemodynamic disturbance (hypertension, hypotension, increased heart rate, nausea, and vomiting) [23, 24], delayed recovery time, prolonged hospital stay, and increased risk of infection [1, 25, 26]. Also, women anxious lose trust in healthcare professionals, and are not satisfied with the care they received, their parents experienced increased costs for unanticipated prolonged hospitalization and treatment of complications [27]. Preoperative anxiety in pregnant women increases the likelihood of bad obstetric outcomes, including delayed breastfeeding and cognitive and neurological impairment in infants, and affects childhood mental health problems [28, 29].

A difficult issue in preoperative care is the presence of preoperative anxiety, previous published evidence suggested that there are certain factors that contribute to higher levels of pre-operative anxiety, including, low socioeconomic status, age, educational level, past surgical exposure, fear of perioperative complications as well as the fear of death from anesthesia or surgery Additionally, patients with chronic medical illnesses, who were worried about their families, and have poor social support increase the odds of preoperative anxiety [3, 30].

There were different previous attempts to prevent and manage preoperative anxiety among surgical patients, including the establishment of preoperative educational interventions and providing non-pharmacological methods such as music therapy, chewing gum [3, 31, 32], spiritual practices, strong social support, acupuncture, and massage [33]. In addition, pharmacological methods with the administration of required medications during the preoperative period appeared to be effective in reducing preoperative anxiety [34].

Though preoperative anxiety is preventable and treatable by perioperative information provision, in southern Ethiopia, two-third (64.3%) of patients get poor quality perioperative information [35], and maternal complications after cesarean section are high (30.1%) at Yirgalem General Hospital [36], and 38.2% in Arba Minch General Hospital [37]. Clinical experiences and studies across the world show that the health of women and fetuses suffers serious consequences due to physiological, pathological, and immunity changes, and women's desire for information about anesthesia and surgeries was not investigated in Ethiopia. Therefore, the purpose of this study was to assess the magnitude of preoperative anxiety and associated factors among women admitted for elective obstetrics and gynecologic surgeries in public hospitals in southern Ethiopia. It contributing to Sustainable Development Goal 3.1 and reducing concerns based on the factors identified in the area.

### Methods and Materials

#### Study area and period

This study was conducted in selected public hospitals of Southern nations’ nationalities and people’s regional states (SNNPR) from May 20^th^, 2022, to June 20^th^, 2022. The SNNPR is one of the eleven regions that were found in Ethiopia; its capital city is Hawassa, which is located around 273 km south of Addis Ababa, the capital city of Ethiopia. Following the separation of the two regional states (Sidama in June 2020 and the Southern West Ethiopian People's Region in 2021), the SNNPR will consist of eleven zones and six special woredas. South Regional Health Bureau reports in 2022 indicate SNNPR has 55 hospitals, 493 health centers, and 2641 health posts, for a total of 45,707 health professionals. All 55 hospitals provide maternal and child health services, including emergency obstetrics and gynecologic-related procedures with anesthesia, but only 11 of them provide major elective obstetrics and gynecologic services. It has an estimated area of 112,343.19 square kilometers, and this region has an estimated density of 132.65 people per square kilometer.

### Study design

An institutional-based cross-sectional study design was conducted.

#### Source population

All women admitted for elective obstetrics and gynecologic surgeries in public hospitals in southern Ethiopia.

#### Study population

All women admitted for elective obstetrics and gynecologic surgeries during the data collection period in selected public hospitals in southern Ethiopia.

#### Inclusion criteria

All women admitted for elective obstetric and gynecologic surgeries.

#### Exclusion criteria

Women with a known psychiatric illness or taking anxiolytic medication.

### Sample Size Determination

The sample size for the primary objective was determined by using the single population proportion formula for a finite population with the assumption of 95% levels of confidence (Z = 1.96), 5% marginal error (d = 0.05), and the prevalence (p) of preoperative anxiety at 63% [20].

$$ni=\frac{Z({\frac{\alpha }{2})}^{2}*p\left(1-p\right)}{{d}^{2}}=\frac{({1.96)}^{2}*0.63\left(1-0.63\right)}{{\left(0.05\right)}^{2}}=358.1\approx 359$$

To determine the sample size for the second objective (associated factors) that affect preoperative anxiety, it was calculated using EPI Info version 7.2 stat calculation by considering 80% power, 95% CI, and the ratio of unexposed-exposed 1 (See Table 1**).**

**Table 1:** Sample size determination for the secondary objective (factors) among women in public hospitals, Southern Ethiopia, 2022

Factors associated with women preoperative anxiety (Reference)	P_1_	P_2_	N
Education [20]	38.1	57.4	253
Previous surgery [20]	34.3	52.8	272

Key: P_1_-percent of exposed with outcome, P_2_- percent of unexposed with outcome N- calculated sample size after added 10% non-response

The sample size determined by the primary objective was greater than that of the second objective (associated factors), so the final sample size was determined by the primary objective, then added a 10% non-response rate, and the final sample size was 395.

### Sampling technique and procedure

**Fig. 1:**
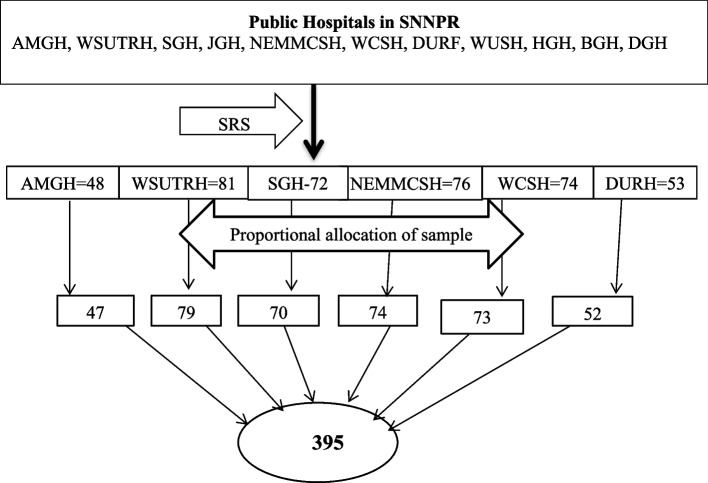
Schematic representation of sampling procedure among women admitted for elective obstetrics and gynecology surgery in public hospitals, Southern, Ethiopia, 2022

In the southern nations, nationalities, and peoples region, there are 11 public hospitals that provide elective obstetrics and gynecologic surgeries. Among those hospitals, six were selected by a simple random sampling method (lottery). From the selected hospitals, eligible women were selected by systematic random sampling after reviewing last year's achievement registration book of elective obstetric and gynecologic surgeries that were performed in each hospital. Consecutively, every admitted woman was included in the study until the final sample size was achieved. Proportional allocation was done to allocate 395 women to each hospital (Fig. 1**).**

Key-SRS-Simple Random Sampling, AMGH-Arba Minch General Hospital, SGH-Sawla General Hospital, JGH-Jinka General Hospital, WSUTRH-Wolayta Sodo University Teaching and Referral Hospital, NEMMCSH-Nigist Eleni Mohammed Memorial Comprehensive Specialized Hospital, WCSH-Worabe Comprehensive Specialized Hospital, WUSH-Wolkite University Specialized Hospital, HGH-Halaba General Hospital, BGH-Butajira General Hospital, DGH-Durame General Hospitals, DURH- Dilla University Referral Hospital.

### Data collection tool and procedures

The data were collected using standardized, validated, and semi-structured questionnaires with interviews the day before surgery after the preoperative evaluation was taken place and the woman was admitted to the ward. The tool was adapted from previous related literature done in Ethiopia [3, 20, 38]. But, the questions which assessed the outcome variable were adopted from standardized, validated, reliable and acceptable tools [39].

The questionnaire contains five sections: socio-demographic characteristics, health status, and clinical characteristics, possible reason for anxiety, and social support assessed by the OSLO social support scale that consists of 3 items with a sum score ranging from 3 to 14 with three categories: poor (3-8), moderate (9-11), and strong (12–14) social support [40].

The outcome variable preoperative anxiety was assessed with APAIS, which is widely used to assess pre-operative anxiety, and consists of six questions that assess three components of anxiety; anesthesia-related anxiety, surgery-related anxiety, and information desire component used and validated in different countries like Sri Lanka [19], and Nepal [18].

APAIS was validated and translated into the Amharic version in Addis Ababa, Ethiopia; which is a reliable and acceptable tool for measuring women’s preoperative anxiety levels and their need for information about anesthesia and surgery with high reliability (Cronbach's alpha coefficient of 0.87), and was well accepted by the study participants, with a 100% response rate with no missing value. The patient chooses the numbers that best describe the intensity of their feelings. Each APAIS item has a weight scale from 1-Not at all to 5-Extremely, the rating of five indicates the presence of high anxiety, and the sum of the scores in all items constitutes the individual’s score [39].

The Pain was assessed with a numeric pain rating scale [41]. Six psychiatric nurses were recruited to collect data and six BSc holder midwives were recruited for supervisory activities, which communicated on a regular basis with data collectors to ensure that the data collection procedure was followed. The questionnaires were prepared first in XLSF format in a Microsoft office excel sheet and then converted to X-form using XLSF online converter to collect the data in open data kit (ODK). ODK collect version 2022.2.3 application was installed on the data collector’s Android mobile phone, to collect the data. Finally, on a weekly basis, the data collector sent the filled questionnaire data to the ODK toolbox server on online connectivity.

### Study variables

#### Dependent variable

Preoperative anxiety.

#### Independent variables

Socio-demographic factors: age, residency, marital status, educational status, occupation, and monthly income.

Social support status, Health status and clinically related factors: types of surgery, parity, chronic medical illness, previous anesthesia and surgery exposure, previous surgical complications, and pain.

Possible reasons for preoperative anxiety: receiving IV fluid, fear of death, unexpected outcome of surgery, concern for family, fear of complications, cosmetics issue, fear of postoperative pain, harm from a medical mistake, need for blood transfusion, fear of the unknown, and inability to recover from anesthesia.

### Operational and term definition’s

Elective surgery( procedure): is done preplanned and performed in advance, does not involve a medical emergency, and is done by the choice of the patient or doctors [42].

The numerical pain rating scale (NRS) is a valid method of pain assessment where patients are asked to score their pain ratings on a scale of 0–10 [41].

Pain score- no pain (0), mild (1-3), moderate (4-6), and severe pain (7-10) [41].

Preoperative anxiety is assessed by APAIS; it was calculated by using five Likert scale-based questions, and the scores were summed by sum scores. Those who scored 11 and above out of 20 were considered to have preoperative anxiety, whereas parents scoring below the 11 were considered to have no preoperative anxiety [11, 39].

High information requirement: On information scale of APAIS, patients scored 8–10 [39].

Average information requirement: on the information scale, patients scored 5–7 [39].

Little or no information requirement: 0n the information scale, patients scored 2–4 [39].

Monthly income: categorized as above the international poverty line (US$1.90) or 97.85 ETB with the current exchange rate multiplied by 30 days = 2935.5 ETB, and below poverty line: (US $1.90) or 97.85 ETB. World Bank Group. Poverty & Equity Brief: Africa, Eastern and Southern, Ethiopia, 2021. www.worldbank.org/poverty.

Social support is classified by sum score as poor (3–8), moderate (9–11), and strong (12–14) social support [40].

### Data quality assurance

To assure the quality of the data, a standardized and validated questionnaire was used to collect the data; a pretest was done on 10% of the sampled population; and training for data collectors was given, including theoretical and practical discussions on ODK data collection techniques. During the data collection, we reviewed the completed questionnaires for critical information before uploading them from the Android mobile phone to the ODK Toolbox server. The completeness and cleanliness of the files that the data collector sent were checked.

### Data processing and analysis

After the data was collected through ODK, it was downloaded as an Excel file, exported to SPSS version 26, checked for completeness, cleaned, and coded. Descriptive statistics were performed to describe the study participant's characteristics. Multi-collinearity was checked by using the variance inflation factor (VIF) to determine if there was correlation between two or more independent variables, and it was tolerated when VIF < 5. The goodness of fit of the model was tested by Hosmer and Lemeshow, and it was found to be 0.17. Both bivariate and multivariable logistic analyses were used to assess the association between each independent variable and the outcome variable.

All variables with a *p*-value < 0.25 at 95% CI in the binary logistic regression model at bivariate logistic regression were transformed into the final multivariable logistic analysis in order to control all potential confounding variables. The odds ratio (OR) with 95% CI was estimated to measure the strength of association factors affecting preoperative anxiety. In this study, a variable with a *p*-value of < 0.05 was considered statistically significant. Finally, the data was organized and presented using text, tables, and figures based on the types of data.

## Results

### Socio-demographic characteristics of study participants

In this study, 395 women were involved, while 389 women participated, with a response rate of 98.48%. The mean age of the study participants was 35.56 ± (SD: 12.2) years, with minimum and maximum ages of 16 and 76, respectively. Among the study participants, 170 (43.7%) were within the age group of 16–31 years. Of the participants, 218 (56.0%) were urban residents. Regarding marital status, 298 (76.6%) were married, 111 (28.5%) attended secondary education, and 142 (36.5%) worked as housewives. From the study participants, 296 (76.1%) had an average monthly income above the poverty line (See Table 2**).**

**Table 2:** Socio-demographic characteristics of women admitted for elective obstetric and gynecologic surgery in public hospitals, Southern Ethiopia, 2022 (*n* = 389)

Variables	Category	Frequency(N)	Percentage (%)
Age in years	16–31	170	43.7
	32–47	165	42.4
	48–63	45	11.6
	64–76	9	2.3
Residence	Urban	218	56.0
	Rural	171	44.0
Marital status	Unmarried	25	6.4
	Married	298	76.6
	Divorced	27	7.0
	Widowed	39	10.0
Educational level	No formal education	99	25.5
	Primary	74	19.0
	Secondary	111	28.5
	Diploma and above	105	27.0
Occupation status	Government employed	66	17.0
	Private employed	89	22.9
	Daily labor	54	13.8
	House wife	142	36.5
	Student	38	9.8
Monthly income	Below poverty line	93	23.9
	Above poverty line	296	76.1

### Factors related to health status and clinical condition

Of a total of 389 women who participated in this study, 203 (52.2%) were admitted for elective obstetric surgery, and 199 (51.2%) of the participants were multiparous (given 2–4 births). Concerning previous anesthesia and surgical exposures, 288 (74%) did not have any previous history of anesthesia or surgery. Of those who had previous exposure to anesthesia and surgery, 23 (22.8%) encountered previous surgery or anesthesia-related complications. 109 (28% of study participants) had a chronic medical illness, and 163 (41.9%) of participants had no preoperative pain (see Table 3**).**

**Table 3:** Factors related with Health status and clinical condition of women admitted for elective obstetric and gynecologic surgery in public hospitals, Southern Ethiopia, 2022

Variables	Frequency(N)	Percentage (%)
**Type of surgery**		
Obstetric	203	52.2
Gynecologic	186	47.8
**Parity**		
Null Para	50	12.9
Prim Para	77	19.8
Multipara	199	51.1
Grand multipara	63	16.2
**Previous anesthesia and surgery**		
Yes	101	26.0
No	288	74.0
**Previous complication**		
Yes	23	22.8
No	78	77.2
**Chronic medical illness**		
Yes	109	28.0
No	280	72.0
**Preoperative pain level**		
No pain	163	41.9
Mild pain	106	27.2
Moderate pain Severe pain	70	18.0
	50	12.9

### Social support status of participants

**Fig. 2:**
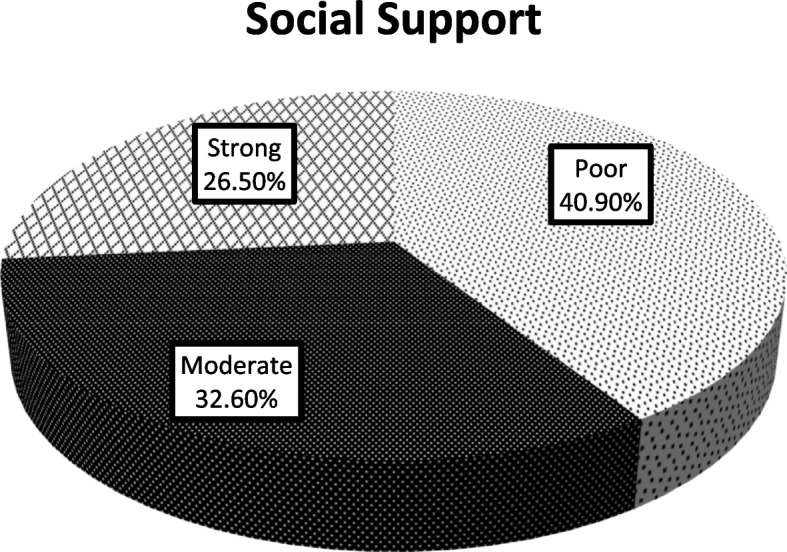
Social support of women admitted for elective obstetric and gynecologic. Surgery public Hospitals, Southern, Ethiopia, 2022

Out of 389 study participants, 159 (40.9%) of participants get poor social support, followed by 130 (33.4%) with moderate social support (see Fig. 2**).**

### Magnitude of preoperative anxiety and desire for information

**Fig. 3:**
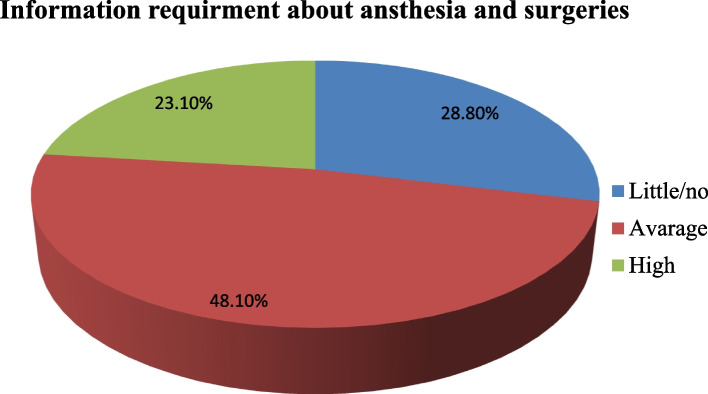
Information requirement about anesthesia and surgeries among women admitted for elective Obstetrics and gynecologic surgery in public Hospitals southern Ethiopia

The magnitude of preoperative anxiety among women admitted for elective OB/GYN surgery in southern Ethiopia was 222 (57.1%) (95% CI = 51.4, 61.5), with an APAIS mean (± SD) score of 12.37 ± (SD = 3.55). The minimum and maximum APAIS scores were 5 and 20, respectively. Regarding information requirements about anesthesia and surgeries, 187 (48.1%) women required an average amount of information, followed by 112 (28.8%) women who required little or no information. The mean score was 6.36 (SD = 2.12), with minimum and maximum scores of 2 and 10, respectively **(**See Fig. 3**).**

### Reasons for preoperative anxiety

**Fig. 4:**
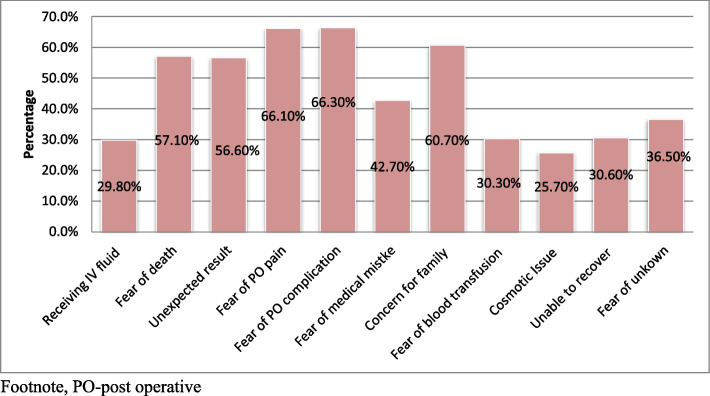
Reasons for preoperative anxiety among women admitted for elective obstetric and gynecologic surgeries in public hospitals, southern Ethiopia, 2022. Footnote, PO-post operative

Among a total of 389 study participants, the most common reasons for preoperative anxiety were fear of postoperative complications 258 (66.3%), post-operative pain 257 (66.1%), concern for family 236 (60.7%), fear of death 222 (57.1%), and an unexpected result of operation 220 (56.6%). Cosmetic issues were the least common reason for preoperative anxiety in this study, accounts 100 (25.7%) (See Fig. 4).

### Factors associated with preoperative anxiety

In binary logistic regression analysis, types of the proposed surgery, previous anesthesia and surgery exposure, chronic medical illness, fear of death, unexpected result of the operation, fear of post-operative pain, fear of post-operative complications, concern for family, social support, and information requirements about anesthesia and surgery were candidates for the final multivariable logistic regression analysis. In the multivariable analysis, type of surgery, previous anesthesia, and surgery exposure, fear of post-operative pain, concern for family, social support, and information requirements about anesthesia and surgeries were statistically associated with preoperative anxiety.

The odds of preoperative anxiety among women admitted for gynecologic surgeries were two times higher than those among women admitted for obstetric surgery (AOR = 2.0, 95% 95%CI = 1.21, 3.32). Women who encountered previous anesthesia and surgery had two times higher odds of preoperative anxiety than women who had no previous exposure to anesthesia and surgeries (AOR = 2.09, 95% CI = 1.10, 3.96). The odds of preoperative anxiety among women who were fear about post-operative pain were 1.9 times higher than who do not (AOR = 1.96, 95% CI = 1.08, 3.53), and the odds of preoperative anxiety among women who were worried about their family were 2.5 times higher than who did not worried about their family (AOR = 2.56, 95%CI = 1.49, 4.37). Women who had poor social support were 3.7 times (AOR = 3.75, 95% CI = 1.99, 7.09) more likely to develop preoperative anxiety, while women who had moderate social support had 3 times higher odds of preoperative anxiety than women with strong social support (AOR = 3.27, 95% CI = 1.74, 6.17). In addition, the odds of preoperative anxiety among women who needed more information about anesthesia and surgeries were 4.6 times higher than among women who required no information (AOR = 4.68, 95% CI = 2.16, 10.13) (See Table 4).

**Table 4:** Bivariate and multivariable analysis of factors affecting preoperative anxiety among women admitted obstetric and gynecologic surgery at hospitals, southern Ethiopia, 2022

Variables	Anxiety		COR(95%CI)	AOR(95%CI)	*P*-value
	**Yes (%)**	**No (%)**			
Type of proposed surgery					
Obstetric	90(44.3)	113(55.7)	1	1	
Gynecologic	132(71.0)	54(29.0)	3.06(2.01–4.67)	2.00(1.21–3.32)	0.007
Previous history of anesthesia					
Yes	76(75.2)	25(24.8)	2.95(1.78–4.91)	2.09(1.10–3.96)	0.024
No	146(50.7)	142(49.3)	1	1	
Chronic medical illness					
Yes	75(68.8)	34(31.2)	1.99(1.25–3.18)	1.45(0.80–2.61)	0.215
No	147(52.5)	133(47.5)	1	1	
Fear of death					
Yes	152(68.5)	70(31.5)	3.00(1.98–4.57)	1.65(0.95–2.84)	0.071
No	70(41.9)	97(58.1)	1	1	
Unexpected result of operation					
Yes	148(67.3)	72(32.7)	2.63(1.74–3.99)	1.14(0.66–1.97)	0.634
No	74(43.8)	95(56.2)	1	1	
Fear post-operative pain					
Yes	177(68.9)	80(31.1)	4.27(2.73–6.68)	1.96(1.08–3.53)	0.025
No	45(34.1)	87(65.9)	1	1	
Fear of complication					
Yes	171(66.3)	87(33.7)	3.08(1.99–4.76)	1.00(0.54–1.81)	0.978
No	51(38.9)	80(61.1)	1		
Concern for family					
Yes	166(70.3)	70(29.7)	4.10(2.66–6.32)	2.56(1.49–4.37)	0.001
No	56(36.6)	97(63.4)	1		
Social support					
Poor	115(72.3)	44(27.7)	5.79(3.37–9.98)	3.75(1.99–7.09)	0.000
Moderate	75(59.1)	52(40.9)	3.20(1.85–5.53)	3.27(1.74–6.17)	0.000
Strong	32(31.1)	71(68.9)	1	1	
Information requirement about anesthesia and surgery					
Little/ no	46(41.1)	66(58.9)	1	1	
Average	101(54)	86(46.0)	1.68(1.04–2.70)	1.61(0.92–2.82)	0.097
High	75(83.3)	15(16.7)	7.17(3.67–14.02)	4.68(2.16–10.13)	0.000

Key- 1-Reference

## Discussions

In this study, the overall magnitude of preoperative anxiety among women admitted for elective obstetrics and gynecologic surgery in Southern Ethiopia was 57.1%( 95%CI = 51.4–61.5). the result was in line with the study's findings of India at 55% [15]. However, the finding is higher than studies conducted in Thailand 23.2% [13], and Seri Lanka 40.6% [19]. This higher level of preoperative anxiety in this study might be due to the low education status of the women, poor quality of preoperative visits and evaluation, and also living in non-civilized countries like Ethiopia, which makes the patient poor access to perioperative information and education makes the patient anxious [35], and also obstetric patients become more anxious due to fear and concern for the unborn fetus in addition to themselves.

In contrast, the magnitude of preoperative anxiety in this study was lower than fining from India at 63.54% [14],Pakistan at 72.7% [17], and Gondar at 63% [20]. This might be due to difference in socioeconomic status, the instrument used, and the time of data collection. For example, higher anxiety in Gonder might be due to the involvement of women who underwent emergency CS who were more anxious than planned surgery. This was supported by a study done in India [15]. It is known that acute types of surgeries make the patients more anxious due to poor anesthetic checks up. This was supported by a study done in Jima which stated that adequate information provided during the preoperative period was one way to prevent preoperative anxiety, but this was impossible at the time of emergency surgeries [38].

This study demonstrated that the odds of preoperative anxiety among women admitted for gynecologic surgeries were two times higher than women admitted for OB surgery. This is in line with study done in India [14]. This higher anxiety in GYN patients might be due to sex hormonal imbalance, post-menopause-related comorbidities, gynecologic surgery was invasive (life-threatening like cancer), and the presence of pain [13], even pain was no association in this study. And also, GYN surgeries made women more anxious due to the involvement of a more sensitive area of the body that involves reproductive tracts, leading to infertile and being in a lithotomy position making them uncomfortable during some procedures [13]. In contrast to this finding, a study done in Pakistan indicated that a higher level of preoperative anxiety was observed in obstetric patients as compared to gynecologic [43].

The current study found that the odds of preoperative anxiety among women who had previous exposure to anesthesia and surgery were 2 times higher than women who had no previous exposure. This finding was supported by studies conducted in Pakistan [17, 18]. This could be due to previous adverse outcomes and postoperative complications, or women who had previous surgery saw post-operative complications and the death of a neighbor in her previous hospital stay [18]. However, this finding contradicts the Ethiopian finding that previous history of anesthesia and surgeries were reduced the level of preoperative anxiety [38].

In this study, women fearing postoperative pain were significantly associated with a higher likelihood of experiencing preoperative anxiety than other women. This result was consistent with a study conducted in Gondar [20]. This may be due to inadequate advice from medical staff regarding the availability of preoperative and postoperative pain management.

Furthermore, women who were worried about their family were at higher odds preoperative anxiety than women who were not. This study was consistent with a study conducted in Ethiopia [3]. This may be because Ethiopian women were family business owners and had primary responsibility for caring for their families and children, and working as a source of income for their families led to financial frustration and fear due to surgery.

The odds of having preoperative anxiety are higher among women who had poor and moderate social support as compared to women having strong social support. The results of this study were supported by other studies conducted in Egypt, and Colombia [30, 44]. This implies that strong social support plays a role in preventing illness, and promoting patient compliance with the given treatment, and reducing financial related anxiety [45]. Lack of social support, on the other hand, has multiple effects on the physiological systems of our body, including the cardiovascular, immune, and endocrine system that makes more anxious [46].

This study suggested that the odds of preoperative anxiety among women who need more information about anesthesia and surgeries were 4.6 times higher than women who required no information. This finding was supported by studies conducted in Addis Ababa, and Nepal Similar to studies conducted in Addis Ababa [39, 44]. This is because patients in need of comprehensive information may ask from non-anesthetist, the Internet, and neighbors with inadequate knowledge of anesthesia, which can lead to misunderstandings and fearful interpretations of surgery [17]. In contrast, a study conducted in Sri Lanka showed no association between preoperative anxiety and need for information about anesthesia or surgery [19].

### Limitation of the study

There may be social desirability bias, and the level of anxiety may differ based on specific times in the preoperative period; hence, it was not addressed. Also, because the study design is cross-sectional, it was difficult to establish a cause-and-effect relationship.

## Conclusions

Preoperative anxiety was often high in the region. Associated factors were the type of surgery, previous anaesthesia and surgery, fear of postoperative pain, fear for family, social support, and a high information need. So the national and regional health bureaus should develop guidelines and implement strategies to reduce women's preoperative anxiety as part of midwifery care. The women should be assessed regularly during the preoperative visits, and appropriate anxiety reduction and information regarding surgery and anaesthesia should be provided. Further researcher would be responsible for assessing coping strategies to reduce and prevent preoperative anxiety.

## Data Availability

The datasets used and/or analyzed during the current study are available from the corresponding author upon reasonable request. Email: aberamamo031@gmail.com.

## Abbreviations

AOR: Adjusted Odds Ratio
APAIS: Amsterdam Preoperative Anxiety and Information Scale
CI: Confidence Interval
COR: Crude Odds Ratio
CS: Cesarean Section
GYN: Gynecology
OB: Obstetrics
ODK: Open Data Kit
SPSS: Statistical Package for Social Science
WHO: World Health Organization
